# Geophagy as a risk factor for Soil-transmitted helminthic infections among pregnant women attending antenatal care at health institutions in Chiro Town, Eastern Ethiopia

**DOI:** 10.1186/s12884-025-08311-7

**Published:** 2025-11-28

**Authors:** Asfaw Mesfin, Mio Ayana, Mestawet Getachew, Zeleke Mekonnen

**Affiliations:** 1 Chiro General Hospital, Oromia Regional State, Eastern, Ethiopia; 2 School of Medical Laboratory Sciences, Institute of Health, Jimma University, Jimma, Ethiopia; 3 School of Pharmacy, Institute of Health, Jimma University, Jimma, Ethiopia

**Keywords:** Pregnant women, Geophagy, Soil-transmitted helminths, Eastern ethiopia

## Abstract

**Background:**

Geophagia is recognized as a habit of compulsive eating of soil and is practiced globally, especially among African societies. It is especially common among pregnant women. This behavior may increase the risk of soil-transmitted helminth (STH) infections, which can adversely affect maternal health. This study aimed to assess the association between geophagy and STH infections, as well as to identify related risk factors among pregnant women attending antenatal care at health institutions in Chiro Town, Eastern Ethiopia.

**Methods:**

A multicenter, cross-sectional study was conducted from July 1 to September 30, 2022, among 404 pregnant women attending antenatal care at three public health facilities in Chiro Town, using a convenience sampling. Data on socio-demographics and geophagy-related factors were collected through a semi-structured questionnaire. Single stool samples were examined for STHs using direct saline wet mount and Kato-Katz methods. Soil samples were collected from geophagous women’s identified sources and analyzed by a concentration technique. Data were analyzed with SPSS v26 using descriptive statistics and logistic regression, with *p* < 0.05 at 95% CI considered significant.

**Results:**

Among the pregnant women studied, 21.5% were infected with STHs, while 16.8% reported practicing geophagy. *A. lumbricoides* (13.4%) and hookworms (4.7%) were the most commonly identified helminths. A total of 45 soil samples were collected from various sources identified by geophagic women; of these, five tested positive for Ascaris eggs and two for hookworm-like larvae. Multivariable analysis showed that geophagy [AOR = 2.9 (95% CI = 1.6–5.4)], lack of hand washing before meals [AOR = 2.5 (95% CI = 1.4–4.6)], and consumption of raw vegetables [AOR = 3.2 (95% CI = 1.5–6.5)] were significantly associated with STH infections. In addition, illiterate women were twice as likely to practice geophagy [AOR = 2.0 (95% CI = 1.1–3.8)].

**Conclusion:**

This study highlights the high prevalence of STH infections among pregnant women and identifies geophagy as a potential risk factor, though reliance on a single stool sample and a less sensitive diagnostic method may have underestimated the true prevalence. To reduce the risk of these infections, it is crucial to promote awareness of the importance of regular handwashing, avoiding the consumption of raw vegetables, and refraining from the practice of geophagy.

**Supplementary Information:**

The online version contains supplementary material available at 10.1186/s12884-025-08311-7.

## Introduction

Geophagy is recognized as a habit of purposive eating of soil or earth materials and is a type of pica, which involves eating non-food substances. Soil eating is commonly practiced by children, individuals with mental disordered, and pregnant and lactating women in low and middle-income countries, especially in African societies [1, 2]. The health benefits and risks of geophagy remain controversial and are not yet fully understood. Individuals consume soil for various reasons, including its taste, smell, and personal preference, as well as for cultural practices, particularly during pregnancy [1]. Notably, nutrient requirements increase substantially during pregnancy [3]. Thus, pregnant women with nutrient deficiency through craving for soil may consider as the way of supplementing deficient nutrients like iron, magnesium, and calcium [4]. Some studies suggest that soil consumption can provide relief from gastrointestinal disturbance and morning sickness, including nausea and vomiting [5, 6]. The protection hypothesis suggests that geophagy protects from harmful toxin and pathogens [5]. However, soil may contain numerous toxic substances and infectious organism [7–9] Research from various fields has highlighted the potential harmful effects of geophagy, including heavy metal poisoning, especially from lead and the risk of soil-transmitted helmith infections (STHs) [2, 10]. Intestinal parasites transmitted through contaminated soil are collectively referred to as STHs). These include hookworms (*Necator americanus* and *Ancylostoma duodenale*), *Ascaris lumbricoides*, and *Trichuris trichiura*. These infections are most prevalent in tropical and sub-tropical regions of the developing world, where poor environmental sanitation and inadequate personal hygiene are prevalent [11]. Globally, STHs have been estimated to infect more than 895 million people, as nearly 447 million, 290 million and 229 million people are infected with *A. lumbricoides*, *T. trichiura* and hookworms, respectively [12]. These parasites are particularly widespread in sub-Saharan Africa (SSA), Latin America and Asia [13]. Soil is an exceptional habitat for these parasites and premature stages of these parasites require a period of development in the soil before they become infective stage. These parasites are transmitted through contaminated food, water, objects and by direct contact with soil, especially when walking bare foot [13].

Pregnant women are particularly vulnerable to the health effects of STHs due to their increased physiological demands and suppressed immunity [3, 14]. Infections caused by STHs, such as hookworm and *Trichuris trichiura*, which may also be acquired through geophagy, can result in chronic blood loss and subsequent iron-deficiency anemia. This condition increases the risk of preterm delivery, low birth weight, and maternal morbidity and mortality [15]. Moreover, evidence indicates that infants born to mothers infected with STHs may experience adverse birth outcomes [16, 17].

Food craving, food aversion and pica are common during pregnancy and are often attributed to hormonal changes [18, 19]. In Ethiopia, the prevalence of geophagy during pregnancy has been reported as 17.4% in Jimma town, Southwest Ethiopia, and 10.3% at Maytsebri Primary Hospital, North Ethiopia [20, 21]. The presence of infective stages of STHs in soil is of significant public health concern, as these parasites-particularly *Ascaris lumbricoides* and *Trichuris trichiura* -are primarily acquired through ingestion of contaminated soil.

Although previous studies have reported an association between geophagy and STH infections among pregnant women in certain regions of Ethiopia [20, 21], none have directly analyzed soil samples (presumably ingested by pregnant women) to confirm the presence or absence of STH eggs or larvae. In the absence of such evidences, establishing a clear link between soil consumption and STH infection remains challenging. Therefore, this study aimed to assess the association between geophagy and STH infections, as well as to identify related risk factors, among pregnant women attending antenatal care (ANC) at Chiro Town Health Institutions (CTHIs), Eastern Ethiopia.

## Materials and methods

### Study design and setting

A multicenter, cross-sectional study was conducted from July 1 to September 30, 2022 among a pregnant women attending ANC service at three public health facilities in Chiro town; namely: Chiro General Hospital (CGH), Family Guidance Association (FGA) Clinic, and Chiro Health Center (CHC). Chiro town is the administrative center of the West Hararghe Zone, Oromia Region, Eastern Ethiopia, which is 326 km away from Addis Ababa, capital city of Ethiopia. The town is located at approximately 9°05’ North latitude and 40°52’East longitude, with an altitude ranging from 1826 to 1950 m above sea level. It has a maximum and minimum temperature of 23 and 12 °C and rainfall of 1800 and 900 mm, respectively.

### Sample size and sampling procedure

Sample size was determined using a single population proportion formula with a 95% confidence interval, assuming a 20% prevalence of geophagia based on a previous study conducted in Jimma [20]. After adding 10% to account for potential non-response, the final sample size was 423. Health facilities providing ANC services were identified, and the total sample was proportionally allocated according to their average monthly caseload: CGH (*n* = 168), FGA (*n* = 147), and CHC (*n* = 108). Finally, a convenience sampling technique was employed to recruit study participants from each facility.

### Data collection and processing

Three trained data collectors were assigned to the selected health institutions, where they identified and enrolled eligible participants (i.e., pregnant women of any gestational age who volunteered to take part and gave written informed consent) until the allocated sample size for each institution was reached. The data collection tool was adapted from earlier studies [7, 20, 21], and data were gathered through face-to-face interviews. Information collected included socio-demographic, obstetric characteristics, geophagic practices, and risk factors associated with STHs. Additionally, observational data such as participants’ fingernail hygiene and footwear use were collected during the interviews.

### Stool specimen collection and processing

The unique identification code was assigned to each study participant’s questionnaire and stool container. Participants were instructed to provide a thumb-sized stool specimen, free from soil contamination, in a labeled, leak-proof stool cup and submit it immediately to the laboratory. Fresh stool samples were then immediately processed using direct wet mount and single Kato-Katz techniques for detection of intestinal parasites, including STHs, by medical laboratory technologists at respective health facilities.

### Soil sample collection

Following the interviews and stool sample collection, data collectors accompanied each geophagous pregnant woman to the specific locations where she reported obtaining soil for consumption. Guided by the participants, soil samples were collected directly from these sources, including excavated soil from fields and riverbanks, as well as mud scraped from house walls. In local markets, participants identified the specific soil they typically purchased and consumed, which was then obtained for analysis. In addition, some women provided soil samples they had previously collected and stored for personal use. This participant‑guided approach increased the likelihood that collected soil samples reflected those actually consumed, although it cannot definitively confirm an exact match. Accordingly, a total of 45 suspected soil samples were collected, with approximately 300 g obtained from each source. All samples were placed in airtight, clearly labeled plastic bags and transported under strict conditions to the CGH laboratory for processing.

### Soil sample processing

Processing soil sample examination method for the detection of STHs eggs/larvae was modified from the environmental protection agency (EPA), which is combination of flotation and sedimentation technique, as applied in Ghana [22]. Briefly, 50 g of homogenized soil were mixed with 500 ml of tap water and 5 ml of 0.1% Tween 40 and left to soak overnight. The following day, the mixture was filtered through a tea strainer into a second container and allowed to settle for two hours. The sediment was then transferred into 15 ml test tubes, suspended in 0.1% Tween 40 and centrifuged for 5 min at 2000 rotations per minute (rpm). After removing the supernatant, the sediment was re-suspended in ZnSO₄ (specific gravity 1.3) and centrifuged for 3 min at 2000 rpm. The supernatant containing the eggs was poured into the beaker, diluted with tap water, and allowed to settle for approximately three hours. After discarding the supernatant, the sediment was transferred to 15 ml centrifuge tube, washed with tap water, and centrifuged at 2000 rpm for 5 min. The resulting sediment was then re-suspended in 10% normal saline and ether, followed by centrifugation at 2000 rpm for 3 min. Finally, after removing the supernatant, the sediment was placed on a microscope slide and examined under a light microscope at 10× and 40× magnifications.

### Statistical analysis

For analysis, coded data were entered to Epi Data version 3.1 and exported to SPSS version 26.0 for statistical processing. Questionnaire responses and laboratory results were summarized using descriptive statistics, including frequencies and percentages. Variables with a *p*-value less than 0.25 in bivariate analysis were included in multivariable logistic regression, to recognizing that factors not significant in univariate analysis may still act as confounders or become significant in the presence of other covariates [23, 24]. Results are presented as odds ratios (OR) with 95% confidence intervals (CI) and statistical significance was set at *p* < 0.05.

## Results

Of the 423 pregnant women enrolled, 404 (95.5%) provided complete responses: 157 (38.9%) from CGH, 143 (35.4%) from FGA and 104 (25.7%) from CHC. The remaining 19 participants were excluded due to incomplete information, such as failure to provide a stool sample. The participants ranged in age from 15 to 39 years, with a mean age of 26.1. Among them, 254 (62.9%) resided in rural areas. The socio-demographic and obstetric characteristics of the study participants are summarized in Table 1.

**Table 1:** Socio-demographic and obstetric characteristics of pregnant women attending antenatal care at three health facilities in Chiro Town, Eastern Ethiopia (*n* = 404)

Characteristics	Categories	Frequency Number (%)
Age Groups	15–19	69 (17.1)
	20–24	113 (28.0)
	25–29	98 (24.3)
	30–34	68 (16.8)
	35–39	56 (13.3)
Marital Status	Married	384 (95.0)
	Unmarried	7 (1.7)
	Widowed	10 (2.5)
	Divorced	3 (0.7)
Residence	Urban	150 (37.1)
	Rural	254 (62.9)
Educational Level	Illiterate	195 (48.3)
	Literate	209 (51.7)
Occupational Status	Housewife	274 (67.8)
	Others	130 (32.2)
Religion	Orthodox	75 (18.6)
	Muslim	279 (69.1)
	Protestant	37 (9.2)
	Catholic	13 (3.2)
Gestational Age	1 st Trimester	27 (6.7)
	2nd Trimester	142 (35.1)
	3rd Trimester	235 (58.2)
Number of Pregnancies	Primigravida	143 (35.4)
	Multigravida	261 (64.6)

### Prevalence of geophagy and associated risk factors among pregnant women

Of the 404 participants 68 (16.8%) and 28 (6.9%) reported consuming soil/earth and other pica type, respectively (Table 3). In bivariate logistic regression analysis, rural residence, illiteracy, occupational status, Muslim religion, third gestational age, and multigravida were significantly associated with geophagy (*p*-value < 0.25), whereas age categories and marital status were not. However, in the multivariable logistic regression model, only educational level remained significantly associated with geophagy (*p* = 0.034). Illiterate pregnant women were twice as likely to practice geophagy compared to literate women [AOR = 2.0; 95% CI: 1.1–3.8]. **(**Table 2**)**.

**Table 2:** Logistic regression analysis of factors associated with geophagy among pregnant women attending antenatal care at three health facilities in Chiro Town, Eastern Ethiopia

Variable	Category	Geophagy (Yes/No)	AOR (95% CI)	*p*-value
Residence	Urban	18/132	Reference	-
	Rural	50/204	1.2 (0.6–2.2)	0.632
Education	Illiterate	46/149	2.0 (1.1–3.8)	0.034^**∞**^
	Literate	22/187	Reference	-
Occupation	Housewife	51/223	Reference	-
	Others	17/113	1.1 (0.5–2.0.5.0)	0.896
Religion	Orthodox	7/68	Reference	-
	Muslim	56/223	1.9 (0.8–7.1)	0.142
	Protestant	4/33	1.2 (0.3–4.6)	0.776
	Catholic	1/12	0.8 (0.1–7.1)	0.805
Gestational Age	1 st trimester	2/25	Reference	-
	2nd trimester	16/126	1.2 (0.3–5.7)	0.819
	3rd trimester	50/185	2.4 (0.5–11.0)	0.248
Number of Pregnancies	Primigravida	19/124	Reference	-
	Multigravida	49/212	1.3 (0.7–2.4)	0.415

*Abbreviations* *AOR *Adjusted odds ratio, *CI* confidence interval, ^**∞**^statistically significant

### Perception of geophagy practice and awareness of the health hazards with geophagy

Study participants reported various reasons for consuming soil/earth. More than half (52.9%) reported consuming soil for its good smell, 15.7% described a persistent personal desire, and 10% reported enjoyment of its taste. Additionally, 17.1% of participants believed that consuming soil helped alleviate nausea and vomiting associated with morning sickness, while 4.3% reported using it as a therapeutic remedy for gastric pain. In contrast, only 4.4% of study participants perceived geophagy as having harmful health effects unrelated to infectious diseases, including STHs. Interestingly, 10.3% of geophagous mothers reported baking the soil before consumption to increase its flavor (Table 3).

**Table 3:** Geophagic practice among pregnant women attending antenatal care at three health facilities in Chiro Town, Eastern Ethiopia

Variables	Categories	Frequency Number (%)
Geophagy	Yes	68 (16.8)
	No	336 (83.2)
Reason for geophagy	Taste	7 (10.0)
	Smelling	37 (52.9)
	Personal interest	11(15.7)
	To avoid morning sickness	12 (17.1)
	Healing	3 (4.3)
Frequency of geophagy	Always	29 (41.4)
	Sometimes	41 (58.6)
Soil color	Black soil	44 (62.9)
	Red soil	15 (21.4)
	White soil	11 (15.7)
Complication after geophagy	Yes (within 24 h)	18 (26.5)
	No	50 (73.5)
Source of ingested Soil	From their farm	36 (52.9)
	House wall	8 (11.8)
	From riverbank	14 (20.6)
	From market	10 (14.7)
Type of other pica consumed	Soft stone	6 (1.5)
	Charcoal	12 (3.0)
	Coffee residue	10 (2.5)
Baking soil before ingestion	Yes	7 (10.3)
	No	61 (89.7)
Awareness about geophagy risk	Yes	3 (4.4)
	No	65 (95.6)

### Prevalence of soil-transmitted helminths among pregnant women

The overall prevalence of STHs among pregnant women, as determined using Kato-Katz method was 21.5% (87/404). The majority of STHs positive cases were infected with *A. lumbricoides* (54; 13.4%), followed by hookworms (19; 4.7%). A total of 5 participants (1.2%) presented with co-infections of *A. lumbricoides* and hookworms. All STH-positive cases were of light infection intensity, with egg counts ranging from 144 to 1464 eggs per gram (EPG) for *A. lumbricoides* (< 4999 EPG), 72–984 EPG for hookworms (< 1999 EPG), and 96–672 EPG for *T. trichiura* (< 999 EPG). Additional intestinal parasites were identified using direct wet mount stool examination method. These included *G. lamblia* (*n* = 20), *E. histolytica/dispar* (*n* = 5), *E. vermicularis* (*n* = 3), *H. nana* (*n* = 2), and *S. mansoni* (*n* = 2). Moreover, two cases of co-infection with *G. lamblia* and *E. histolytica/dispar* were observed.

### Factors associated with soil-transmitted helminthic infections

In the analysis, factors such as age groups, occupational status, hand washing after toilet using water and soap, fingernail trimming habit, eating unwashed raw vegetables/fruits, source of water for domestic use and presence of domestic animal at their homes were not significantly associated with STH infections based on bivariate logistic regression (p-value > 0.25). However, variables such as rural residence, being illiterate, trimester stage, geophagia, absence of hand washing habit before meal and after contact with soil, absence of using latrine, presence of dirty material under fingernail, eating of uncooked vegetables and shoe wearing behavior were significantly associated with STH infections in the bivariate logistic regression analysis (p-value < 0.25) (Supplementary file, Table 5 and Table 6). Subsequently, those all variables which had significant association during bivariate logistic regression analysis were entered into multivariate logistic regression model. The results indicated that geophagy practice, lack of hand washing habit before meal using soap and water, and eating habit of raw vegetables were significantly associated with STH infections among pregnant women (p-value < 0.05) (Table 4). Multivariate analysis showed that pregnant women who did not practice handwashing before meals had significantly higher odds of STH infection compared to those who did [AOR = 2.5; 95% CI: 1.4–4.6; *p* < 0.05]. Similarly, pregnant women who consumed raw vegetables had 3.2 times higher odds of STH infection compared to those who did not [AOR = 3.2, 95% CI: 1.5–6.5]. (Table 4)

**Table 4:** Logistic regression analyses of factors associated with soil-transmitted helminths among pregnant women attending antenatal care at three health facilities in Chiro Town, Eastern Ethiopia

Variables	*STHs* (Yes/No)	AOR (95% CI)	*p*-value
Residence			
Urban	25/125	Reference	-
Rural	62/192	1.1 (0.6–2.1)	0.717
Education			
Literate	38/171	Reference	-
Illiterate	49/146	1.0 (0.6–1.9)	0.913
Gestation			
1st	3/24	Reference	-
2nd	25/117	1.6 (0.4–5.9)	0.508
3rd	59/176	2.3 (0.6–8.5)	0.201
Geophagy			
Yes	29/39	2.9 (1.6–5.4)	0.001^∞^
No	58/278	Reference	-
Hand wash before meal			
Yes	51/260	Reference	-
No	36/54	2.5 (1.4–4.6)	0.003^∞^
Latrine			
Yes	78/299	Reference	-
No	9/18	0.9 (0.4–2.5)	0.901
Dirt under fingernail			
Yes	58/170	1.3 (0.7–2.2)	0.378
No	29/147	Reference	-
Eating raw vegetables			
Yes	77/216	3.2 (1.5–6.5)	0.002^∞^
No	10/101	Reference	-
Shoes wear			
Yes	55/251	Reference	-
No	32/66	1.3 (0.7–2.5)	0.392
Hand wash after contact with soil			
Yes	48/222	Reference	
No	39/95	1.3 (0.7–2.3)	0.437

*Abbreviations* *AOR * Adjusted odds ratio, *CI* confidence interval, ^∞^statistically significant

### Detection of soil-transmitted helminths eggs/larvae from suspected soil samples

Geophagic women were getting soil from different places according to their preferences. Based on their information, a total of 45 suspected soil samples were collected and analyzed. Of the 45 soil samples examined, five soil samples tested positive for *Ascaris* and two were positive for larvae resembling hookworm larvae and none were positive for *Trichuris.*

## Discussion

Soil-transmitted helminths has significant public health importance with more than 895 million people infected in the globe [12]. Nearly 250 million girls and adult women are living in endemic area for STHs [13]. In SSA, an estimated that 37.7 million women of reproductive age are infected with hookworms, of whom nearly 6.9 million are pregnant women [25]. These infections can affect both the mother and the developing fetus potentially lowering hemoglobin levels and impacting fetal growth, as well as influencing the immune systems of both mother and baby [3, 14]. Hence, infection with STHs during pregnancy may present problem like iron deficiency anemia [26], which might resulted in maternal morbidity and mortality [15]. As a result, there was an evidence that the infant born from STH infected mothers encounters some problem such as: decreased infant birth weight, child growth retardation, premature birth [27, 28]. Ethiopia is one of the mostly afflicted countries by STHs and immune-modulation that happened during pregnancy enhances their vulnerability to STH infections [14].

This study found an overall STH prevalence of 21.5% among pregnant women which is consistent with previous findings from Jimma town health institutions, Southwest Ethiopia 20% [20], Shahura Primary Hospital, Northwestern Ethiopia 19.8% [29], and a Tertiary provincial Hospital, Philippines 20.7% [30]. However, this prevalence is lower than reports from Maytsebri primary hospital, North Ethiopia 51% [21] and Gilgel Gibe Dam area, Southwest Ethiopia 41% [31]. The differences in prevalence rates between these studies possibly attributed to differences in diagnostic methods, study periods, altitude and weather condition of the study area, sanitation infrastructure, and hygiene practices. In contrast, it is higher than the 13.7% reported in Kenya [32] and 12% reported in Benin [27], which may reflect variations in socio-demographic and behavioral factors. According to World Health Organization (WHO), preventive deworming for pregnant women is recommended when the prevalence of hookworm and/or *T. trichiura* is 20% or higher because of their strong link to anemia [13]. In this study, the overall prevalence of STH infections was 21.5%, which meets the threshold for intervention. However, *A. lumbricoides* was the most commonly detected species, while hookworm was less frequent and *T. trichiura* was not found at all. This suggests that although deworming is recommended, the specific pattern of infection in this setting may call for a more targeted approach. Since *A. lumbricoides* is less strongly linked to anemia compared to hookworm and *T. trichiura*, it’s important that control programs consider the local context alongside global guidelines to ensure the best possible outcomes.

Geophagy is a common practice during pregnancy, and in this study, 16.8% of pregnant women attending ANC at CTHIs reported deliberately consuming soil. According to the participants, this behavior was often driven by the smell or taste of soil, personal cravings, the belief that it helps relieve morning sickness (nausea and vomiting), and for perceived medicinal benefits recognized within the society. This habit may be partly due to a lack of awareness about the potential health risks associated with soil consumption. Our findings also showed that illiteracy was significantly associated with geophagy, which is consistent with a study from Mashau Village in Limpopo Province, South Africa [33]. This suggests that women with lower levels of education may be less aware of the possible harms of geophagy or may not perceive it as a risky behavior. Geophagy can begin at any trimester of pregnancy, but in our study, more than half of the women reported starting the practice during the second trimester. This aligns with findings from other studies in sub-Saharan Africa [7, 34]. One possible explanation is that physiological changes during this period such as the increased need for iron due to red blood cell production and fetal growth [3] may trigger cravings that lead to soil ingestion [34].

The health impact of geophagy remains controversial and uncertain, as literature recorded both positive and negative health effects. Some research suggests that geophagy may provide essential nutrients such as calcium and magnesium, offer protection against pathogens and toxins, and help relieve gastrointestinal upset [2, 4, 35]. However, it also poses significant public health concerns as the soil may contain toxic substances like lead, cadmium, mercury, and arsenic. More importantly, it can carry parasites that cause STH infections [2]. Although the health effects of geophagy remain a subject of ongoing debate, our study identified it as a significant risk factor for STH infections. This finding aligns with studies conducted in other regions of Ethiopia [20, 21], and may be explained by the direct ingestion of soil contaminated with parasite eggs or larvae, which increases the likelihood of infection. Specifically, we observed a strong association between geophagy and infections with *A. lumbricoides* and hookworm, but not with *T. trichiura*. This result is consistent with a study conducted in a humid tropical region of Nigeria [7]. Furthermore, the prevalence of *A. lumbricoides* was significantly higher among women who practiced geophagy compared to those who did not, mirroring results reported in another Nigerian study [36].

During interviews, many participants reported consuming soil without any form of treatment, potentially exposing them to STHs [36, 37]. In this study, parasitological analysis of soil samples consumed by pregnant women revealed the presence of STH eggs primarily *A. lumbricoides* which supports the idea that geophagy may increase the risk of infection. This finding is consistent with studies from Nigeria, where helminth eggs or larvae were also identified in geophagic soil samples [7, 37]. However, it should be noted that the soil examined in the present study or other studies may not directly correspond to the soil actually consumed, representing a limitation in definitively proving this association. On the other hand, some studies from other parts of sub-Saharan Africa did not detect any STH eggs or larvae in soils suspected to be contaminated [38, 39]. In our case, although *T. trichiura* is known to be transmitted through contaminated soil, we did not detect its eggs in any of the samples analyzed. This could be due to environmental factors such as soil composition, temperature, humidity, and sunlight exposure, all of which influence the survival and detectability of *Trichuris* eggs [40]. Another possibility is that the sampling or detection methods used may not have been sensitive enough to identify low level contamination. Further research using more varied sampling approaches may help clarify these results.

Our study also found that not washing hands with soap and water before meals significantly increases the risk of STH infections. This agrees with findings from Maytsebri Primary Hospital in Northern Ethiopia [21] and Shahura Primary Hospital in Northwestern Ethiopia [29]. These results emphasize the crucial role of proper handwashing in preventing the oral transmission of parasites. Additionally, consuming raw vegetables was associated with higher odds of STH infections among pregnant women, which is consistent with research from Mecha district, Northwest Ethiopia [41]. This may be because raw vegetables can act as mechanical vectors for helminth eggs [42], and if not properly cooked or cleaned, these eggs can be ingested, leading to infection.

### Strength and limitations

This study has notable strengths and limitations. A key strength is the analysis of soil to investigate the presumed source of geophagy, offering insights into a potential route of STH transmission. However, the soil analyzed may not directly correspond to that actually consumed, limiting the ability to definitively link geophagy to infection. The cross-sectional design restricts causal inference and temporal assessment of risk factors. Convenience sampling, used due to time and resource constraints, may limit representativeness; future studies with probability-based sampling could improve generalizability. Only a single stool sample was collected per participant, potentially underestimating the true prevalence due to variability in egg shedding, whereas multiple samples would enhance diagnostic sensitivity. Finally, hemoglobin levels were not assessed, precluding evaluation of the possible relationship between anemia, geophagy, and STH infections. Incorporating anemia screening in future research would provide a more comprehensive understanding of these interactions.

## Conclusions

This study demonstrated a considerable prevalence of STHs among pregnant women, with *A. lumbricoides* most common, followed by hookworms and *T. trichiura*. Key risk factors included geophagy, poor hand hygiene, and consumption of raw vegetables, with illiteracy strongly associated with geophagy. The true prevalence may be underestimated due to reliance on a single stool sample and the limited sensitivity of diagnostic methods. These findings highlight the need for health education during antenatal care, promoting safe food practices, handwashing, and avoidance of soil consumption. Routine STH screening and deworming after the first trimester should also be considered, and further research is warranted to clarify the links between geophagy, helminth infection, and anemia. Moreover, this study revealed that the prevalence of geophagy is considerable, highlighting the need to assess its broader health impacts beyond STH infections and to develop targeted preventive interventions.

## Supplementary Information

Supplementary material 1.

Supplementary material 2.

## Data Availability

The dataset supporting the findings of this study will be available from the first author upon reasonable request.

## Abbreviations

CGH: Chiro General Hospital
CHC: Chiro Health Center
CTHIs: Chiro Town Health Institutions
FGA: Family Guidance Association
STHs: Soil-transmitted Helminthic Infections
ANC: Antenatal Care
SSA: Sub-Saharan Africa
WHO: World Health Organization
EPG: Eggs Per Gram
EPA: Environmental Protection Agency
