# Supplementary material for: Geophagy as a risk factor for Soil-transmitted helminthic infections among pregnant women attending antenatal care at health institutions in Chiro Town, Eastern Ethiopia
Source: BMC Pregnancy Childbirth. 2025 Nov 28;25:1277. doi: 10.1186/s12884-025-08311-7 (PMC12661869; doi:10.1186/s12884-025-08311-7)
Supplement: Supplementary file 1 — Supplementary material 1. [file 12884_2025_8311_MOESM1_ESM.docx]

**Annex-V: Questionnaire (English version)**

Jimma University, Institute of Health, School of Medical Laboratory Sciences for the study of geophagy as risk behavior for soil-transmitted helminth infections and associated risk factors among pregnant women at Chiro Health Institutions, from July 01 to September 30, 2022, Ethiopia.

I kindly request you to give appropriate response for each question. Your response will be kept confidential.

Participants’ ID Number: ------------------------

Name of health facility............

**Part one: Socio-demographic and obstetric information**

1. Age of participant----------------- (In year)

2. Marital status of the participant: a) Married b) Unmarried c) Widowed d) Divorced e) others-----------

3. Residence: a) Urban b) Rural

4. Educational level: a) Literate b) Illiterate

5. Occupational: a) Housewife b) Merchants c) Government employee d) daily laborer e) Student f) Other (specify)………………

6. Religion: a) Orthodox b) Muslim c) Protestant d) Catholic e) Other (specify)

7. Gestational age in week -----------------

8. Number of pregnancies (live birth and still birth) -------------

**Part two: Geophagic practice**

9. Do you have habit of soil consumption during this pregnancy? a) Yes b) No (If No go to question number 22)

10. If Q# 9 yes, how often do you consume soil? a) Always b) Sometimes

11. When do you start eating soil? A) Before pregnancy b) at first trimester c) at second trimester d) at third trimester

12. Do you prepare/process soil for consumption? a) Yes b) No

13. If Q # 12 yes, what processing method is used? -------------------------------------

14. What is the reason behind for processing soil? -------------------------------------------

15. What is the reason behind to eat soil? a) Taste b) Smell c) medicinal d) to avoid nausea/vomiting e) personal interest f) Other (specify) --------------------

16. Can you tell me types of soil consumed? a) Black soil b) Loam (reddish soil) c) Chalky (whitish soil) d) Other (specify) -----------

17. Do you experience any complication after eating soil? (Within 24 hours) a) Yes b) No

18. If Q# 17 yes, what types of complication you encounter? a) Constipation b) Nausea c) Vomiting d) Abdominal pain e) Hearth burning f) Other (specify) --------

19. Do you know the risk faced by soil ingestion? a) Yes b) No

20. If Q# 19 yes, can you mention them? ---------------------------

21. Do you have the specific site where that kind of soil is obtained? a) excavated from known area b) River bank c) House wall d) from farm f) market g)Other (specify) ------

22. Do you ever consume any another non-food items? a) Yes b) No

23. If Q # 22 yes, can you call the type of non-food substance consumed? a) Soft stone b) Charcoal c) Coffee residue d) Other (specify)----------------------

**Part three: Associated risk factors for soil transmitted helminths**

24. Do you have the habit of hand washing before meal using soap and water? a) Yes b) No

25. Do you use a latrine? a) Yes b) No

26. Do you have the habit of hand washing after toilet using soap and water? a) Yes b) No

27. Is there any dirty material in your finger nail? (Observe it) a) Yes b) No

28. Do you have fingers nail trimming habit? a) Yes b) No

29. Do you have a habit of eating unwashed raw vegetables/fruits? a) Yes b) No

30. Do you have a habit of eating undercooked vegetables/fruits? a) Yes b) No

31. Do you wash your Hand after contact with soil? a) Yes b) No

32. Which source of water you use for drinking? a) Tap b) Stream/river/lake c) Other (specify)_____________

33. Which source of water you use for washing utensils? a) Tap b) Stream/river/lake c) Other (specify)_____________

34. Do you have a habit of shoe wearing? a) Yes b) No

45. Do you have close contact with domestic animals? a) Yes b) No

Name of the Interviewer: ________________________Signature and Date: _____________

Checked by P. Investigator: _______________________ Signature: ___________

Thanks!!!
