# Supplementary material for: Geophagy as a risk factor for Soil-transmitted helminthic infections among pregnant women attending antenatal care at health institutions in Chiro Town, Eastern Ethiopia
Source: BMC Pregnancy Childbirth. 2025 Nov 28;25:1277. doi: 10.1186/s12884-025-08311-7 (PMC12661869; doi:10.1186/s12884-025-08311-7)
Supplement: Supplementary file 2 — Supplementary material 2. [file 12884_2025_8311_MOESM2_ESM.docx]

Table 5: Bivarate and multivariate logistic regression analyses of factors associated with geophagy among pregnant women attending antenatal care at three health facilities in Chiro Town, Eastern Ethiopia

| Variable | Category | Geophagy (Yes/No) | COR (95% CI) | *p-value* | AOR (95% CI) | *p-value* |
| --- | --- | --- | --- | --- | --- | --- |
| Residence | Urban | 18/132 | Reference | - | Reference | - |
|  | Rural | 50/204 | 1.8 (1.1-3.2) | 0.048**^∞^** | 1.2 (0.6-2.2) | 0.632 |
| Education | Illiterate | 46/149 | 2.6 (1.5-4.6) | 0.001**^∞^** | 2.0 (1.1-3.8) | 0.034**^∞^** |
|  | Literate | 22/187 | Reference | - | Reference | - |
| Occupation | Housewife | 51/223 | Reference | - | Reference | - |
|  | Others | 17/113 | 0.7 (0.4-1.2) | 0.167**^∞^** | 1.1 (0.5-2.0) | 0.896 |
| Religion | Orthodox | 7/68 | Reference | - | Reference | - |
|  | Muslim | 56/223 | 2.4 (1.1-5.6) | 0.036**^∞^** | 1.9 (0.8-7.1) | 0.142 |
|  | Protestant | 4/33 | 1.2 (0.3-4.3) | 0.805 | 1.2 (0.3-4.6) | 0.776 |
|  | Catholic | 1/12 | 0.8 (0.1-7.2) | 0.850 | 0.8 (0.1-7.1) | 0.805 |
| Gestational Age | 1^st^ trimester | 2/25 | Reference | - | Reference | - |
|  | 2^nd^ trimester | 16/126 | 1.6 (0.3-7.3) | 0.554 | 1.2 (0.3-5.7) | 0.819 |
|  | 3^rd^ trimester | 50/185 | 3.4 (0.8-14.8) | 0.105**^∞^** | 2.4 (0.5-11.0) | 0.248 |
| Number of Pregnancies | Primigravida | 19/124 | Reference | - | Reference | - |
|  | Multigravida | 49/212 | 1.5 (0.9-2.7) | 0.161**^∞^** | 1.3 (0.7-2.4) | 0.415 |

Abbreviations: COR= crude odds ratio; AOR = Adjusted odds ratio; CI=confidence interval; ^∞^statistically significant

Table 6: Bivarate and multivariate logistic regression analyses of factors associated with soil-transmitted helminthes among pregnant women attending antenatal care at three health facilities in Chiro Town, Eastern Ethiopia

| Variables | STHs (Yes/No) | COR  (95% CI) | *p-value* | AOR  (95% CI) | *p-value* |
| --- | --- | --- | --- | --- | --- |
| Age groups |  |  |  |  |  |
| 15-19 | 14/55 | Reference. | - | - | - |
| 20-24 | 26/87 | 1.2 (0.6-2.4) | 0.668 | - | - |
| 25-29 | 17/81 | 0.8 (0.4-1.8) | 0.630 | - | - |
| 30-34 | 15/53 | 1.1 (0.5-2.5) | 0.800 | - | - |
| 35-39 | 15/41 | 1.4 (0.6-3.3) | 0.393 | - | - |
| Occupational status |  |  |  |  |  |
| Housewife | 62/212 | 1.2 (0.7-2.1) | 0.348 | - | - |
| Others | 25/105 | Reference | - | - | - |
| Residence |  |  |  |  |  |
| Urban | 25/125 | Reference | - | Reference | - |
| Rural | 62/192 | 1.6 (1.0-2.7) | 0.069^∞^ | 1.1 (0.6-2.1) | 0.717 |
| Education |  |  |  |  |  |
| Literate | 38/171 | Reference | - | Reference | - |
| Illiterate | 49/146 | 1.5 (0.9-2.4) | 0.091^∞^ | 1.0 (0.6-1.9) | 0.913 |
| Gestation |  |  |  |  |  |
| 1^st^ | 3/24 | Reference | - | Reference | - |
| 2^nd^ | 25/117 | 1.7 (0.5-6.1) | 0.410 | 1.6 (0.4-5.9) | 0.508 |
| 3^rd^ | 59/176 | 2.7 (0.8-9.2) | 0.118^∞^ | 2.3 (0.6-8.5) | 0.201 |
| Geophagy |  |  |  |  |  |
| Yes | 29/39 | 3.6 (2.0-6.2) | 0.001^∞^ | 2.9 (1.6-5.4) | 0.001^∞^ |
| No | 58/278 | Reference | - | Reference | - |
| Hand wash before meal |  |  |  |  |  |
| Yes | 51/260 | Reference | - | Reference | - |
| No | 36/54 | 3.2 (1.9-5.4) | 0.001^∞^ | 2.5 (1.4-4.6) | 0.003^∞^ |
| Latrine |  |  |  |  |  |
| Yes | 78/299 | Reference | - | Reference | - |
| No | 9/18 | 1.9 (0.8-4.4) | 0.128^∞^ | 0.9 (0.4-2.5) | 0.901 |
| Hand washing after defecation |  |  |  |  |  |
| Yes | 63/241 | Reference | - | Reference | - |
| No | 24/76 | 1.2 (0.7-2.1) | 0.490 | - | - |
| Dirt under fingernail |  |  |  |  |  |
| Yes | 58/170 | 1.7 (1.1-2.8) | 0.031^∞^ | 1.3 (0.7-2.2) | 0.378 |
| No | 29/147 | Reference | - | Reference | - |
| Fingernail trimming |  |  |  |  |  |
| Yes | 49/163 | 1.2 (0.8-1.9) | 0.419 | - | - |
| No | 38/154 | Reference | - | - | - |
| Eating unwashed raw vegetables/fruits |  |  |  |  |  |
| Yes | 55/207 | 0.9 (0.6-1.5) | 0.720 | - | - |
| No | 32/110 | Reference | - | - | - |
| Eating raw vegetables |  |  |  |  |  |
| Yes | 77/216 | 3.6 (1.8-7.3) | 0.001^∞^ | 3.2 (1.5-6.5) | 0.002^∞^ |
| No | 10/101 | Reference | - | Reference | - |
| Shoes wear |  |  |  |  |  |
| Yes | 55/251 | Reference | - | Reference | - |
| No | 32/66 | 2.2 (1.3-3.7) | 0.002^∞^ | 1.3 (0.7-2.5) | 0.392 |
| Source of water for domestic use |  |  |  |  |  |
| Pipe | 46/177 | Reference | - | - | - |
| Unprotected | 41/140 | 1.1 (0.7-1.8) | 0.623 | - | - |
| Hand wash after contact with soil |  |  |  |  |  |
| Yes | 48/222 | Reference | - | Reference |  |
| No | 39/95 | 1.9 (1.2-3.1) | 0.010^∞^ | 1.3 (0.7-2.3) | 0.437 |
| Presence of domestic animal |  |  |  |  |  |
| Yes | 27/84 | 1.2 (0.7-2.1) | 0.402 | - | - |
| No | 60/233 | Reference | - | - | - |

**Abbreviations: COR= crude odds ratio; AOR = Adjusted odds ratio; CI=confidence interval; ^∞^statistically significant**
